# Involvement of miRNAs in Metabolic Herbicide Resistance to Bispyribac-Sodium in *Echinochloa crus-galli* (L.) P. Beauv.

**DOI:** 10.3390/plants11233359

**Published:** 2022-12-02

**Authors:** Carlo Maria Cusaro, Carolina Grazioli, Enrica Capelli, Anna Maria Picco, Marta Guarise, Enrico Gozio, Pietro Zarpellon, Maura Brusoni

**Affiliations:** 1 Department of Earth and Environmental Sciences, University of Pavia, Via S. Epifanio 14, 27100 Pavia, Italy; 2 Agricola 2000 S.c.p.A., Via Trieste 9, 20067 Tribiano, Italy

**Keywords:** herbicide resistance, bispyribac-sodium (ALS-inhibitor), *Echinochloa crus-galli* (L.) P. Beauv., epigenetic regulation, miRNAs, Cytochrome P450, glutathione-S-transferase, eIF4B

## Abstract

Several mechanisms involved in weed herbicide resistance are unknown, particularly those acting at the epigenetic level, such as the capacity of small-non-coding RNAs (sncRNAs) to target messenger RNAs of genes involved in herbicide detoxification. The transcription of these sncRNAs is stimulated by epigenetic factors, thereby affecting gene expression. This study was carried out in order to evaluate, for the first time in *Echinochloa crus-galli* (L.) P. Beauv. (barnyardgrass), the capacity of miRNAs to regulate the expression of genes associated with bispyribac-sodium detoxification. The expression profiles of eight miRNAs with a high degree of complementarity (≥80%) with mRNAs of genes involved in herbicide detoxification (CYP450, GST and eIF4B) were determined by qRT-PCR before and after herbicide spraying. Five of the miRNAs studied (gra-miR7487c, gma-miR396f, gra-miR8759, osa-miR395f, ath-miR847) showed an increased expression after herbicide application in both susceptible and resistant biotypes. All the miRNAs, except gra-miR8759, were more highly expressed in the herbicide-resistant biotypes. In specimens with increased expression of miRNAs, we observed reduced expression of the target genes. The remaining three miRNAs (ata-miR166c-5p, ath-miR396b-5p and osa-miR5538) showed no over-expression after herbicide treatment, and no difference in expression was recorded between susceptible and resistant biotypes. Our results represent a first overview of the capacity of miRNAs to regulate the expression of genes involved in bispyribac-sodium detoxification in the genus *Echinochloa.* Further research is required to identify novel miRNAs and target genes to develop more focused and sustainable strategies of weed control.

## 1. Introduction

Herbicide resistance (HeR) is a major threat to worldwide agricultural systems. HeR is an example of the adaptive evolution of weeds in response to human selective pressures, resulting in the evolution of global resistance to a wide range of herbicides in many weed species [1,2]. Generally, weed resistance to herbicides consists of two main mechanisms: target site resistance (TSR) and non-target site resistance (NTSR). TSR involves DNA mutation of genes expressing herbicide target proteins, causing a reduction in affinity and efficacy of the herbicide for its target site. NTSR mechanisms involve metabolic processes of detoxification that are able to decrease the amount of herbicide that can reach target organelles in the plant (i.e., vacuolar sequestration or enzymatic degradation of herbicide molecules) [1,2,3,4,5,6,7]. Chemical control in the form of herbicides has so far represented the most effective tool for managing weeds.

As a result of strict European regulations on the use of plant protection products (Reg EC/1107/2009), the repeated use of an increasingly narrow range of herbicides targeting the same metabolic pathways has led to the evolution of herbicide-resistant populations [1,2,3,4,5,6,7,8,9,10,11,12].

Furthermore, artificial selection of agronomic traits in rice (*Oryza sativa* L.) has unintentionally promoted the evolution of crop-like weed biotypes. As a result, the weeds can evade chemical control and eradication from fields, allowing them to spread throughout the agroecosystem (*Vavilovian mimicry*) [13,14]. 

*Echinochloa* species are the most prevalent weeds infesting crop cultivations and paddy fields globally due to their wide ecological success and ability to mimic the crop. Among them, *Echinochloa crus-galli* (L.) P. Beauv. (barnyardgrass) is one of the most problematic and widespread species in agriculture. It is listed as a major weed species in Italian rice fields and has developed resistance to several classes of herbicides [15,16,17].

*E. crus-galli* is an allo-hexaploid (2n = 6x = 54) annual plant characterized by high genetic variability and intraspecific polymorphism, making its morphological identification difficult. It produces a large number of seeds, is highly competitive, has a large adaptive capacity and is resistant to several herbicide classes, all features that can lead to a reduction in agricultural productivity and make it difficult to control [18,19,20,21,22,23,24,25,26]. Worldwide losses in rice yield due to *E. crus-galli* are estimated to be around 35% of the total crop [15,16,27]. Hence, understanding the mechanisms concerning the adaptability and occurrence of herbicide resistance in this weed is essential for establishing adequate, effective and sustainable weed management strategies. 

Although both TSR and NTSR have been widely studied, NTSR mechanisms are more complex to explain and investigate [28,29,30,31,32,33]. Previous works on *E. crus-galli* have mainly focused on the mechanisms underlying NTSR, but not on the epigenetic processes that regulate the genes involved in herbicide detoxification [28,29,30,31]. These latter mechanisms have been poorly studied in this species [15]. 

A recent study conducted on resistant lines of *Echinochloa colona* (L.) Link in Western Australia suggested there are unknown mechanisms of herbicide resistance [34]. It has been hypothesized that, in addition to DNA mutations or indels in TSR- or NTSR-related genes, herbicide resistance might also be influenced by epigenetic processes such as DNA and histone modifications, and various non-coding RNAs, particularly microRNAs (miRNAs) [32,33].

MicroRNAs are small, endogenous, non-coding RNAs (sncRNAs), 20–24 nucleotides in length, that have been shown to regulate post-transcription gene expression. They function by pairing with the 3′ UTR of target mRNAs and repressing translation, or by targeting the mRNA for degradation [35,36,37,38,39,40,41,42]. If it is partially complementary to the miRNA, the mRNA is targeted for translational inhibition. In this way, an individual miRNA can post-transcriptionally affect the expression of hundreds of mRNAs, profoundly altering both qualitative and quantitative gene expression [42]. Cleavage of mRNA appears to be the predominant mechanism of miRNA-driven regulation in plants. Furthermore, miRNAs are conserved across species and kingdoms. For example, it has been reported that plants and animals share miRNAs of the miR854 family, indicating a common origin as regulators of transcriptional mechanisms. Trans-kingdom miRNA conservation has also been highlighted between fungal miRNA-like RNAs (milRNAs) and plant and animal miRNAs, which show many similarities [43,44,45]. These small RNAs play an important regulatory role in various biological processes of plants. Their spectrum of action is extremely wide and most miRNAs do not function independently but are involved in overlapping regulatory networks. They act as epigenetic regulators to control gene expression of key enzymes involved in multiple plant metabolic pathways. For example, by regulating proteins critical for development and growth, including those involved in xenobiotic detoxification, they negatively regulate the target mRNA at the post-transcriptional level without modifying the gene sequences [35,36,37,38,39,40,41,42,43,44,45]. It is known that miRNAs also play a crucial role in regulating plant adaptive responses to biotic and abiotic stresses and help restore cell homeostasis upon sudden environmental changes. Biotic or abiotic stresses cause plants to over- or under-express miRNAs or to synthesize new miRNAs, which in turn control the expression of genes involved in various stress response pathways [46,47,48,49,50].

NTSR due to herbicide detoxification represents the most common mechanism that allows plants to overcome chemical control. Cytochrome P450 monooxygenases (also called CYP or P450) and glutathione S-transferases (GST) represent the main enzymes acting in these processes. The family of Cytochromes P450 encodes heme-dependent enzymes that normally catalyze oxygen and NADPH-dependent monooxygenation reactions. The P450 family includes multiple genes which facilitate the denaturation of a wide range of chemicals. The GST gene family includes multifunctional enzymes that catalyze the conjugation of glutathione into various substrates to form a polar S-glutathionylated (R-SG) product. The R-X substrates that are conjugated are often hydrophobic and electrophilic toxic chemicals, including herbicides. The diversity of the GST gene family allows them to detoxify a wide range of chemicals and to play a role in the synthesis of several secondary metabolites. The involvement of both gene families in response to herbicide application and resistance has been widely documented. The expression and regulation of these genes play fundamental roles in herbicide resistance [5,7,51]. 

Nevertheless, the regulation of enzymes involved in herbicide detoxification by miRNAs remains unclear and is an under-researched area in *E. crus-galli*. To the best of our knowledge, there have been no studies on the regulatory mechanisms of herbicide resistance mediated by miRNAs, although the role of miRNAs in regulating plant responses to abiotic and biotic stresses is well understood [46,47,48,49,50]. The only study which has analyzed the regulation of genes involved in resistance to fenoxaprop-P-ethyl by miRNAs was conducted by Pan et al. (2016) on *Beckmannia syzigachne* (Steud.) Fernald [52]. 

This research was carried out as a part of the *EpiResistenze* research project (https://epiresistenze.unipv.it/, accessed on 6 November 2022) aimed at analyzing the epigenetic mechanisms involved in herbicide resistance in the genus *Echinochloa* P. Beauv., in order to support more effective weed prevention and control programs.

In this study, a set of eight genes (cytochrome P450 monooxygenase, glutathione-S-transferase and eIF4B translation initiation factor), which have previously been found to be involved in the herbicide detoxification network in many plant species, was selected to be analyzed. Some of these genes have already been described in *E. crus-galli* (*CYP81A68, GSTF1, EcGST* and *eIF4B*), while others have been described in *Echinochloa phyllopogon* (Stapf) Stapf ex Kossenko (*CYP72A122, CYP72A254, CYP71AK2* and *CYP81A22*) [3,4,30,53,54]. The eight miRNAs used in this study were identified in silico by bioinformatic analysis, based on a high degree of complementarity with the mRNA sequences of the genes considered. The expression profile of miRNAs and their gene target was assessed in herbicide-susceptible and -resistant barnyardgrass biotypes before and after herbicide administration by means of quantitative RT-PCR (qRT-PCR). 

The purpose of this research was to assess the role of miRNAs in the regulation of the expression of genes involved in bispyribac-sodium detoxification in *E. crus-galli* from rice fields in the Lombardy region of northern Italy. Furthermore, we evaluated if transcription of miRNAs is triggered by herbicide administration.

## 2. Results

The sensitivity and resistance of plants to bispyribac-sodium were tested three weeks after herbicide treatment, and resistant (R) and susceptible (S) biotypes were identified.

**Figure 1:**
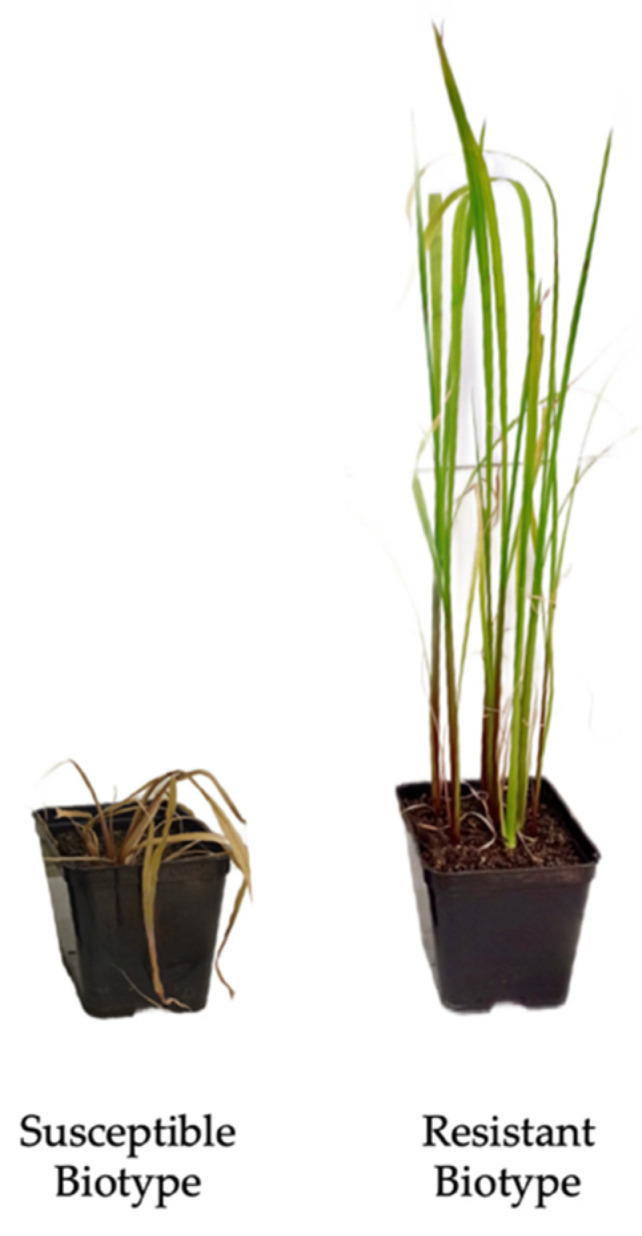
*Echinochloa crus-galli* susceptible and resistant biotypes observed three weeks after treatment with bispyribac-sodium. Wilting of the susceptible plant leaves is clearly visible.

Figure 1 shows an example of a susceptible and a resistant barnyardgrass biotype three weeks after treatment with bispyribac-sodium.

The expression levels of eight genes known to be involved in herbicide detoxification and of eight miRNAs selected for their ability to target the same genes were analyzed. The miRNAs were first subjected to extensive bioinformatic analysis using the psRNATarget: A Plant Small RNA Target Analysis Server [55,56]. This tool allowed us to determine the degree of complementarity between the miRNA and the mRNA of putative target genes: miRNAs with a proportion of nucleotide correspondence ≥80% were selected for this study. 

The miRNAs selected have not previously been tested in *E. crus-galli*. 

Table 1 lists the miRNAs and genes considered.

**Figure 2:**
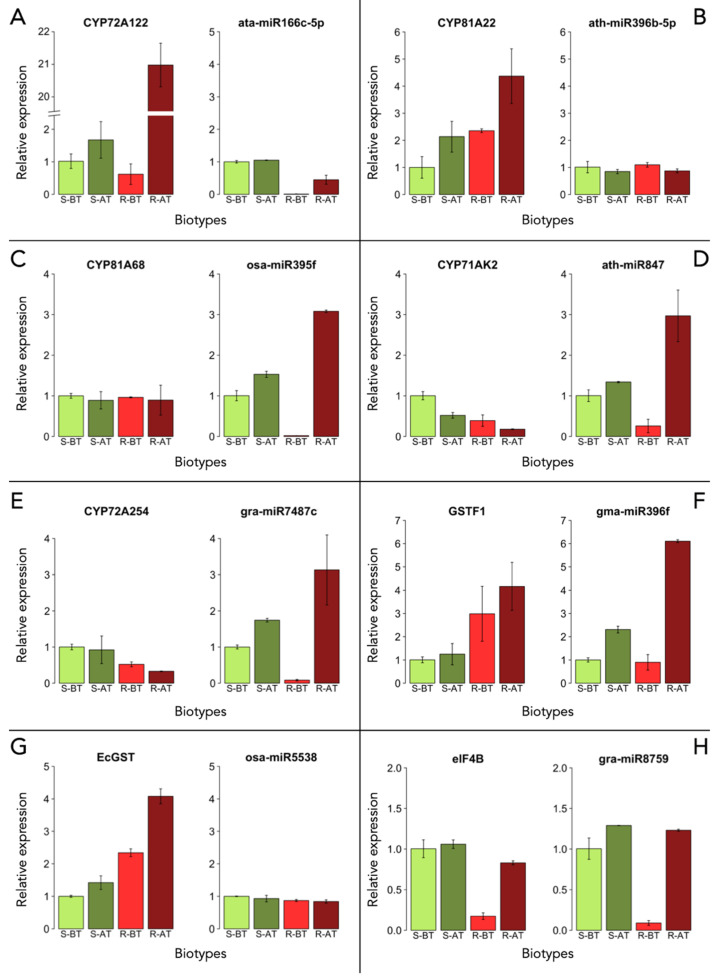
Expression levels of the miRNAs studied and the mRNA of their target genes in susceptible (S) and resistant (R) specimens of Echinochloa crus-galli before (BT) and after (AT) bispyribac-sodium application. (**A**) *CYP72A122* and ata-miR166c-5p; (**B**) *CYP81A22* and ath-miR396b-5p; (**C**) *CYP81A68* and osa-miR395f; (**D**) *CYP71AK2* and ath-miR847; (**E**) *CYP72A254* and gra-miR7487c; (**F**) *GSTF1* and gma-miR396f; (**G**) *EcGST* and osa-miR5538; (**H**) *eIF4B* and gra-miR8759. Error bars indicate the standard deviation of three replicates.

The expression analysis of both miRNAs and their target genes was assessed by quantitative REAL-TIME PCR (qRT-PCR) (see Section 4) [57]. Results are reported in Figure 2. The expression levels of miRNAs and their target genes are compared before (BT) and after (AT) bispyribac-sodium application in susceptible (S) and resistant (R) specimens of *E. crus-galli*. 

In Figure 2A, the values of expression of the *CYP72A122* gene and ata-miR166C-5p are reported. The expression of *CYP72A122* increased after treatment with bispyribac-sodium in both susceptible and resistant biotypes. The highest expression value was recorded in resistant biotypes, with a value 20 times higher than the untreated susceptible specimens (1.01 ± 0.22 vs. 20.97 ± 1.67; *p* < 0.05). The expression of ata-miR166C-5p showed a slight increase in resistant biotypes after treatment (0.01 ± 0.006 vs. 0.45 ± 0.13; *p* < 0.05); however, it was found to be under-expressed when compared to the susceptible biotypes.

Figure 2B shows the values of expression of the *CYP81A22* gene and of ath-miR396b-5p. In resistant biotypes, *CYP81A22* expression was significantly higher (*p* < 0.05) than in the sensitive biotypes both before and after treatment, as expected due to the detoxifying role of the protein. Moreover, we observed that bispyribac-sodium application was able to trigger the expression of *CYP81A22*. In both susceptible and resistant biotypes, *CYP81A22* expression doubled after herbicide administration (from 1.00 ± 0.39 to 2.13 ± 0.56; from 2.35 ± 0.06 to 4.37 ± 1.00; *p* < 0.05). No difference in the expression of ath-miR396b-5p was recorded either before or after bispyribac-sodium application for both susceptible and resistant biotypes.

Figure 2C highlights that the CYP81A68 gene had similar expression values in both susceptible and resistant biotypes before and after treatment. osa-miR395f expression appeared to be induced by bispyribac-sodium in susceptible specimens (S-AT) with an increase of around 50% (from 1.00 ± 0.12 to 1.53 ± 0.07; p < 0.05). In resistant biotypes before treatment (R-BT), the miRNA expression was negligible (0.02 ± 0.001), but after herbicide application (R-AT), expression significantly increased (3.08 ± 0.03) compared to the reference sample (S-BT) (p < 0.05). 

Figure 2D shows that CYP71AK2 expression appeared to be affected by bispyribac-sodium application, as observed by a reduction in the expression values of around half in both the susceptible and resistant biotypes (p < 0.05). The transcription of ath-miR847 appeared to be stimulated by herbicide application, with a slight increase in the susceptible biotypes (from 1.00 ± 0.14 to 1.34 ± 0.01) and a larger increase in the resistant biotypes (from 0.25 ± 0.16 to 2.96 ± 0.63; p < 0.05).

The same trend was observed in the CYP72A254 gene and gra-miR7487c, as shown in Figure 2E. For susceptible and resistant biotypes, higher gene expression values were recorded before treatment and there was a further reduction after herbicide application. For gra-miR7487c expression, herbicide treatment stimulated the expression of this miRNA, with a significant increase in the resistant biotypes (from 0.08 ± 0.02 to 3.13 ± 0.96; p < 0.05), tripling in expression when compared to the susceptible specimens before treatment (S-BT).

In Figure 2F, GSTF1 was over-expressed by three times in resistant biotypes compared to susceptible biotypes before bispyribac-sodium application, and over-expressed by four times in resistant biotypes after herbicide spraying (p < 0.05). The expression of gma-miR396f significantly increased only after treatment in both susceptible and resistant biotypes, with the highest increase observed in resistant biotypes, from 0.89 ± 0.33 to 6.10 ± 0.05 (p < 0.05). 

Figure 2G shows that EcGST expression was higher in resistant biotypes before bispyribac-sodium administration (R-BT) in comparison to susceptible biotypes. The application of herbicide induced a further stimulation of EcGST expression in both susceptible and resistant biotypes. A significant increase was observed in the resistant biotype (from 2.34 ± 0.12 to 4.08 ± 0.23; p < 0.05), about four times higher compared to the reference untreated specimen (S-BT). The expression of osa-miR5538 was the same across all specimens before and after herbicide application. 

In Figure 2H, the expression of the translation initiation factor eIF4B was significantly lower in the resistant biotypes compared to the susceptible biotypes before bispyribac-sodium administration (p < 0.05). After treatment, there was no significant increase in eIF4B expression in the susceptible biotypes (S-AT). In contrast, in the resistant biotypes, expression increased fourfold upon treatment (from 0.17 ± 0.04 to 0.83 ± 0.02); however, this expression value was lower than that observed in the reference untreated susceptible biotypes (S-BT). Before herbicide application, the expression of gra-miR8759 was around eight times lower in resistant (R-BT) compared to susceptible (S-BT) biotypes. Herbicide treatment induced an increase in the expression of gra-miR8759 in both biotypes, with comparable values (1.29 ± 0.002 in S-AT and 1.23 ± 0.01 in R-AT).

## 3. Discussion

In this study, we investigated the role of miRNAs in the regulation of genes involved in the detoxification of bispyribac-sodium (Nominee^®^). The expression of a set of miRNAs (ata-miR166c-5p, ath-miR396b-5p, osa-miR395f, ath-miR847, gra-miR7486c, gma-miR396f, osa-miR5538 and gra-miR8759), targeting five different cytochrome P450 genes (*CYP72A122, CYP81A22, CYP81A68, CYP71AK2* and *CYP72A254*), two glutathione-S-transferase genes (*GSTF1* and *EcGST*) and the eIF4B translation initiation factor, was evaluated in susceptible and resistant *E. crus-galli* biotypes from rice fields in the Lombardy region of northern Italy. These genes have previously been found to be involved in herbicide resistance [3,4,15,30,54]. The set of miRNAs was selected based on their ability to pair with mRNA sequences of the above-mentioned genes, following a bioinformatic analysis using the psRNATarget: A Plant Small RNA Target Analysis Server [55,56]. This study was conducted as part of a research project (*EpiResistenze*) which aimed to investigate the role of epigenetic mechanisms in the occurrence and regulation of herbicide resistance. 

To date, studies have primarily analyzed the role of DNA mutations (TSR) or genes that detoxify herbicides (NTSR) in resistant weeds [9,10,28,29,30,31,34,51,54], but the role of epigenetic factors is still poorly understood. 

Pan et al. (2022) demonstrated that different expression values of *CYP81A68* in resistant and susceptible *E. crus-galli* Chinese plants are related to different levels of methylation in the promoter region of the gene [15]. Limited information is available on the role of miRNAs in the adaptation of weeds against chemical control and in the occurrence of herbicide resistance. Pan et al. (2016) analyzed differential regulation of some miRNAs in fenoxaprop-P-ethyl-resistant *B. syzigachne*, highlighting the regulatory role of bsy-miR160a-5p, bsy-miR164a, bsy-miR408-3p, novel-bsy-miR-12, novel-bsy-miR-15, novel-bsy-miR-19 and novel-bsy-miR-29 on stress response genes related to NTSR [52]. 

The only study on barnyardgrass which considered miRNAs was carried out by Fang et al. (2015), who found an increase in the expression of some miRNAs in response to phenolic acids produced by *O. sativa* (rice allelopathy) [58]. 

To our knowledge, this study is the first focusing on the role of miRNAs in the regulation of genes involved in herbicide resistance in the genus *Echinochloa*.

The miRNAs we considered in this study have previously been identified in other plant species as playing a role in hybridization [59], growth regulation [60,61,62,63] and in response to various biotic and abiotic stresses including nutrient deficiency, drought, cold and salinity [64,65,66,67,68,69,70]. Moreover, miRNAs of the miR396 family are known to target oxidases, including cytochromes involved in xenobiotic detoxification [71].

Through bioinformatic analysis using the psRNATarget tool, the ability to couple mRNAs of the selected target genes and to down-regulate their expression was verified in silico (see Table 1).

From our results, five of the miRNAs analyzed (osa-miR395f, ath-miR847, gra-miR7487c, gma-miR396f and gra-miR8759) showed an increased expression after herbicide treatment in both susceptible and resistant biotypes. Except gra-miR8759, these miRNAs recorded the highest degree of expression in the resistant biotypes, and we observed a reduced expression of the corresponding target proteins. The remaining three miRNAs (ata-miR166c-5p, ath-miR396b-5p and osa-miR5538) showed no over-expression after herbicide treatment and no differences in expression between susceptible and resistant biotypes. 

These results indicate that the miRNAs analyzed in this study play a role in the regulation of plant response to bispyribac-sodium treatment. Herbicide application triggered the transcription of miRNAs which down-regulated the expression of target genes, reducing their detoxification ability. In instances when herbicide spraying did not stimulate the transcription of miRNAs, the target gene mRNAs could be translated into proteins, leading to herbicide detoxification.

**Figure 3:**
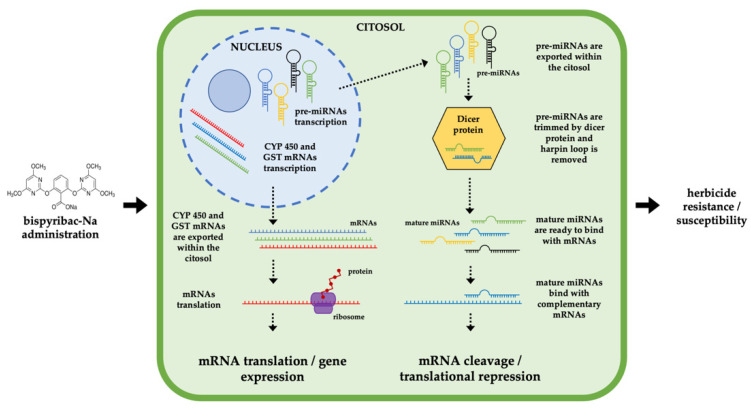
The role of miRNAs in the adaptive response of plants to bispyribac-sodium.

These findings suggest there is involvement of the selected miRNAs in the occurrence of Nominee^®^ resistance/susceptibility in *E. crus-galli* from Italian rice paddy fields. Figure 3 summarizes how miRNAs regulate the adaptive response of plants to bispyribac-sodium. 

The results of this work are of great interest as they represent the first report on the regulation of herbicide resistance by miRNAs in the genus *Echinochloa*. They also verify the expression of miRNAs in *E. crus-galli*, as has been previously described in other species. This latter observation corresponds with recent reports that these molecules are conserved not only within a species but also across kingdoms, influencing gene expression even in phylogenetically unrelated organisms [72,73,74,75,76]. 

The topic of regulatory networks in miRNAs has been scarcely investigated. Considering that epigenetic mechanisms are triggered by various abiotic and biotic ecological factors, plant response to stresses (including herbicides) may involve complex physiological pathways where environmental signals stimulate many responses, some of which can affect the expression of genes involved in herbicide metabolism. 

It is important to underline that translational down-regulation due to miRNAs occurs at the post-transcriptional level and represents a further step in the control of gene expression that can lead to a reduction in, or lack of, the gene product. The assessment of the entire miRNAoma of *E. crus-galli* is critical to better understand the regulation of proteins involved in herbicide resistance and the interaction with ecological factors in this noxious weed. Moreover, it would be useful to identify the ecological factors able to regulate miRNAs in the agricultural environment. A thorough understanding of miRNA regulation of detoxifying genes, taking into account epigenetic–environment interactions, will help to optimize precision weed management (PWM) technologies. More targeted and sustainable strategies for controlling herbicide resistance could help to reduce chemical inputs, improving food health and protecting the environment.

## 4. Materials and Methods

### 4.1. Plant Materials, Growth Conditions and Herbicide Treatment

Seeds were collected from adult plants of E. crus-galli in experimental parcels from paddy fields in the Lombardy region of northern Italy. Resistant (R) biotypes that had survived chemical control were identified in a parcel in which bispyribac-sodium (Nominee^®^) had been applied. Susceptible (S) biotypes were identified from a non-treated parcel and were therefore used as the reference susceptible line.

Seeds from each biotype were sown in separate 100 mL pots containing a universal organic compound (Vigorplant Italia S.r.l., Fombio, Italy). Plants were maintained in a growth chamber with a mean temperature of 20 °C and 70% relative humidity and a photoperiod of 14/10 h (day/night). Three biological replicates were used for each treatment.

Bispyribac-sodium (Nominee^®^) was sprayed at the label dose of 60–75 mL/with the addition of adjuvant (Biopower^®^ 1 L/ha). Chemical treatment was applied to the entire plant collection at the three-leaf stage using a Honda WJR 2525 ET^®^ backpack sprayer (Honda Motor Co., Ltd., Minato, Tokyo, Japan) with a spray pressure of 4 bar and speed of 43 mL/s, resulting in a spray volume of 300/400~L/ha. 

The sensitivity to bispyribac-sodium herbicide was tested three weeks after treatment following European and Mediterranean Plant Protection Organization (EPPO) standards (EPPO, 2011) [77]. Plant tissue was collected before treatment at the three-leaf stage from susceptible (S-BT) and resistant (R-BT) biotypes. After herbicide administration, tissues were re-collected from susceptible (S-AT) and resistant (R-AT) biotypes. Leaves were stored at −40 °C until RNA extraction.

The presence of mutations in the acetolactate synthetase gene known to be involved in Target Site Resistances (TSRs) against bispyribac-sodium was tested through selective amplification and digestion of the ALS gene in order to select only wild-type barnyardgrass plants [28]. 

### 4.2. RNA Extraction

Total RNA was extracted from leaf tissues of susceptible (S) and resistant (R) biotypes before and after herbicide spraying using the RNeasy Plant Kit (QIAGEN SpA, Hilden, Germany) according to the manufacturer’s instructions. 

### 4.3. Candidate NTSR Gene Selection and Candidate miRNA Prediction

The metabolic genes to be tested were selected on the basis of previously published studies and by using a specific scoring database (Table 2). The expressions of cytochrome P450 monooxygenase (CYP81A68, CYP71AK2, CYP72A122, CYP72A254, CYP81A22) and gluthatione-S-transferase (EcGST, GSTF1) genes were analyzed. In addition, the expression of the eIF4B gene was assessed. This gene is present in eukaryotic organisms and is involved in the detoxification of the xenobiotics pathway [30].

The primer sequences of candidate non-target site resistance (NTSR) genes were designed using the program Primer BLAST (https://www.ncbi.nlm.nih.gov/tools/primer-blast/index.cgi, accessed on 8 May 2022) from the most conserved region of each sequence obtained in GenBank (http://www.ncbi.nlm.nih.gov/genbank, accessed on 4 May 2022 ). For each gene, primer pairs were designed based on an annealing temperature of around 60 °C, with a size of approximately 20 bp and an expected amplification fragment of 100–150 bp (Table 2). 

A set of miRNAs able to target the genes selected for the study were searched on the basis of complementarity scoring and secondary structure analysis between the sequence of mRNA target genes and the sequence of putative miRNA, using the psRNATarget: A Plant Small RNA Target Analysis Server tool (https://www.zhaolab.org/psRNATarget/home, accessed on 1 June 2022 ) [55,56]. miRNAs with complementarity ≥80% were chosen for the analyses. 

The expression of genes and miRNAs was assessed through quantitative real-time PCR (qRT-PCR) [57]. 

In Table 2 and Table 3, the genes and miRNAs, together with respective primer sequences, are listed.

### 4.4. cDNA Synthesis and qRT-PCR Analysis

cDNA was obtained following a reverse transcription reaction with the miRCURY LNA RT Kit (QIAGEN SpA). The reaction mixture contained 10 μL of RNA template (5 ng/µL), 4 μL of 5 × miRCURY RT reaction buffer, 2 µL of 10 × miRCURY RT enzyme mix and 10 µL of nuclease-free H_2_O. The reverse transcription reaction was performed at 37 °C for 60 min, followed by 95 °C for 10 min.

The expression profiles of cytochromes P450, gluthatione-S-transferase and eIF4B genes were determined using qRT-PCR. The amplification was carried out using the SYBR Green^®^ kit (Takara Holdings Inc., Shimogyō-ku, Kyoto, Japan), with the Applied Biosystems 7300 Real-Time PCR System (Thermo Fisher Scientific, Waltham, MA, US) on 96-well plates (PCR-96M2-HS-C^®^, Axygen Scientific – part of Thermo Fisher Scientific, Waltham, MA, US). For each sample, amplification was carried out in a total volume of 10 μL containing 2 μL of DNA sample (~5 ng/µL) and 8 μL of master mix composed of 5 μL of TB Green Prmix Ex Taq (Tli RNaseH Plus, Takara Holdings Inc., Shimogyō-ku, Kyoto, Japan), 0.5 μL (10 μM) of forward and reverse primers, 0.5 μL of ROX Reference Dye and 1.5 µL of nuclease-free H_2_O. The amplification reactions were run in a three-step program with an initial incubation at 95 °C for 30 s, followed by 40 cycles of amplification (95 °C for 5 s, 60 °C for 30 s). A dissociation cycle was carried out at 95 °C for 15 s, 60 °C for 1 min, 95 °C for 15 s then increasing the temperature stepwise by 0.3 °C. 

The expression analysis of miRNAs was carried out in a total volume of 10 μL containing 2 μL of 1:80 diluted cDNA sample and 8 μL of master mix composed of 5 μL of TB Green Prmix Ex Taq (Tli RNaseH Plus) (Takara), 1 µL of miRCURY LNA miRNA (QIAGEN SpA) and 2 µL of nuclease-free water. The amplification reactions were run in a three-step program including melting curve analysis with an initial incubation at 95 °C for 30 s, followed by 40 amplification cycles (95 °C for 5 s, 60 °C for 31 s). A dissociation cycle was carried out at 95 °C for 15 s, 60 °C for 1 min, 95 °C for 15 s, then increasing the temperature stepwise by 0.3 °C.

The threshold values (Ct) were determined by the 7300 Real-Time PCR System on-board software. The comparative Ct method (2^−ΔΔCt^ method) was used to calculate the expression levels of candidate genes and of miRNAs [78]. Each sample were tested in triplicate.

The gene-specific primers and miRNAs used for real-time PCR are listed in Table 2 and Table 3.

The determination of relative expression was performed considering b-actin housekeeping gene (HQ395760.1) as an internal reference for protein expression and U6 small nuclear RNA (AT3G14735.1) as an internal reference for miRNAs.

### 4.5. Statistical Analysis

The calculation of relative expression levels was carried out using the ΔΔCt method [78].

The relative expression was calculated by the ΔCt method using Equation (1):

ΔΔCt = (Ct_target_ − Ct_reference_) − (Ct_calibrator_ − Ct_reference_), (1)

where susceptible biotypes (EcgS) were considered as a calibrator [78].

The expression levels of genes and miRNAs calculated for susceptible (S) and resistant (R) barnyardgrass biotypes were presented as means and standard deviations calculated from three replicates.

The relative expression values (fold change) and standard deviations of candidate genes and miRNAs were graphed as bar plots in R 3.6.3 software [79].

Significant differences in expression levels of candidate metabolic genes and relative miRNAs before and after treatment were analyzed using a t-test in R 3.6.3 software [79].

## 5. Conclusions

Our findings highlight the post-transcriptional regulation of cytochromes *P450*, glutathione-S-transferase and *eIF4B* genes by miRNAs triggered by bispyribac-sodium application in *E. crus-galli* Italian biotypes. When the miRNA is over-expressed, it exhibits a negative regulatory function towards the gene target, inducing herbicide susceptibility. Otherwise, the under-expression of the miRNA leads to the occurrence of resistance due to herbicide detoxification. Increased expression after herbicide administration in susceptible and resistant biotypes was recorded for five of the miRNAs studied (gra-miR7487c, gma-miR396f, gra-miR8759, osa-miR395f, ath-miR847). These miRNAs, with the exception of gra-miR8759, were more highly expressed in the herbicide-resistant biotypes. There was no over-expression after herbicide treatment and no differences in expression between susceptible and resistant biotypes for the remaining three miRNAs (ata-miR166c-5p, ath-miR396b-5p and osa-miR5538). In the specimens with high expression values of miRNAs, reduced expression of the target genes was observed.

MicroRNAs previously described in other plant species were selected on the basis of having a high complementarity with target mRNAs of proteins known to be involved in bispyribac-sodium detoxification and previously untested in *E. crus-galli.* The results obtained here represent a preliminary step to better understand the role of epigenetic regulation driven by miRNAs in herbicide resistance. Further analysis will be necessary to expand the known number of miRNAs involved in these metabolic pathways. Despite growing evidence of a central regulatory role by miRNAs in gene expression, these small molecules and their functions are still poorly understood. A deeper knowledge of the plant miRNAoma could be useful to understand how the resistance/susceptibility of weeds to chemical control is influenced by the complex network in which genes and miRNAs synergistically act.

## Figures and Tables

**Table 1:** miRNAs analyzed in *Echinochloa crus-galli* and their target genes.

miRNAs		Target Genes	
Name	a.n. (miRbase)	Name	a.n. (NCBI)
ata-miR166c-5p	MIMAT0037248	*CYP72A122*	AB734013.1
ath-miR396b-5p	MIMAT0000945	*CYP81A22*	AB872310.1
osa-miR395f	MIMAT0000974	*CYP81A68*	OK483200.1
ath-miR847	MIMAT0004278	*CYP71AK2*	AB733990.1
gra-miR7486c	MIMAT0034235	*CYP72A254*	AB755796.1
gma-miR396f	MIMAT0021069	*GSTF1*	HF548530.1
osa-miR5538	MIMAT0022174	*EcGST1*	JX518596
gra-miR8759	MIMAT0034189	*eIF4B1*	AB720070.1

*ata*—*Aegilops tauschii* Coss.; *ath*—*Arabidopsis thaliana* (L.) Heynh.; *gma*—*Glycine max* (L.) Merr.; *gra*—*Gossypium raimondii* Ulbr.; *osa*—*Oryza sativa* L.; *a.n.*—accession number.

**Table 2:** Nucleotide sequences of the primers used for qRT-PCR analysis of CYP450, GST and eIF4B genes’ expression in *Echinochloa crus-galli*.

Gene ID	NCBI a.n.	Primer Sequence (5′-3′)	Reference
*CYP71AK2*	AB733990.1	F: acgtgtgggacaagttcctg	Iwakami et al., 2013 [3]
		R: ggctttgatgcgatcgtctg	
*CYP72A254*	AB755796.1	F: ttacgaggtactccggctgt	Iwakami et al., 2013 [3]
		R: gtcagggtcgtggtgaatgt	
*CYP72A122*	AB734013.1	F: agttcaagccggagaggttc	Iwakami et al., 2013 [3]
		R: catcttggcttcaagcagcg	
*CYP81A68*	OK483200.1	F: gactattcaacccgggcgat	Pan et al., 2022 [15]
		R: caagttctgcacggcaagag	
*CYP81A22*	AB872310.1	F: cggcgcgctggtccagtt	Iwakami et al., 2014 [4]
		R: tgacatgagcagttccatcg	
*EcGST1*	JX518596	F: gccgaggaggacctgaagaac	Li et al., 2013 [54]
		R: gtgactcacagataggcttaccgt	
*GSTF1*	HF548530.1	F: tgcctcttcaaccccatgat	Dalazen et al., 2018 [30]
		R: aggtactcgtgctgggagag	
*eIF4B1*	AB720070.1	F: cgagcagcttacaagggact	Dalazen et al., 2018 [30]
		R: gtggttccataccaccacga	
*b-actin*	HQ395760.1	F: gtgctgttccagccatcgttcat	Li et al., 2013 [54]
		R: ctccttgctcatacggtcagcaata	

**Table 3:** Sequences of mature miRNAs selected for the expression analysis in *Echinochloa crus-galli*.

Name	miRbase a.n.	miRNA Sequence (5′-3′)	Reference
ata-miR166c-5p	MIMAT0037248	ggaacguuggcuggcucgagg	Jia et al., 2013 [59]
ath-miR396b	MIMAT0000945	uuccacagcuuucuugaacuu	John-Rohades et al., 2004 [66]
ath-miR847	MIMAT0004278	ucacuccucuucuucuugaug	Rajagopalan et al., 2006 [67]
gma-miR396f	MIMAT0021069	agcuuucuugaacuucuuaugccua	Radwan et al., 2011 [68]
gra-miR7486c	MIMAT0034235	uuuguccacgugaacagaaaacgc	Xue et al., 2013 [62]
gra-miR8759	MIMAT0034189	ugguggaaguauugugcccgg	Xue et al., 2013 [62]
osa-miR395f	MIMAT0000974	gugaauuguuugggggaacuc	John-Rohades et al., 2004 [66]
osa-miR5538	MIMAT0022174	acugaacucaaucacuugcugc	Wei et al., 2011 [69]
U6 snRNA	NR141593.1	cttcggggacatccgataaaattg	Salanoubat et al., 2000 [70]

*ata*—*Aegilops tauschii* Coss., *ath*—*Arabidopsis thaliana* (L.) Heynh, *gma*—*Glycine max* (L.) Merr., *gra*—*Gossypium raimondii* Ulbr., *osa*—*Oryza sativa* L., *a.n.*—accession number

## Data Availability

Data are contained within the article.
